# Latent Profile Analysis of Occupational Stress and Psychological Capital and the Dual Mechanisms of Psychological Capital in Healthcare Workers’ Sleep Quality

**DOI:** 10.3390/healthcare13233109

**Published:** 2025-11-28

**Authors:** Sijia Yang, Boya Zhang, Jian Chen, Jiahui Li, Bo Zhang, Zhijun Zhou

**Affiliations:** 1 MOE Key Laboratory of Public Health Safety, NHC Key Laboratory of Health Technology Assessment, School of Public Health, Fudan University, No. 130 Dong’an Road, Shanghai 200032, China; yangsijia@scdc.sh.cn (S.Y.); 22111020059@m.fudan.edu.cn (B.Z.); 2 Shanghai Municipal Center for Disease Control and Prevention, No. 1399 Shen Hong Road, Minhang District, Shanghai 200336, China; chenjian_3@scdc.sh.cn (J.C.); lijiahui@scdc.sh.cn (J.L.); 3 Huangpu District Center for Disease Prevention and Control, Huangpu District Health Supervision Institute, 309 Xietu Road, Shanghai 200023, China

**Keywords:** occupational stress, psychological capital, sleep quality, healthcare workers, latent profile analysis, mediation, moderation

## Abstract

**Background**: Sleep disorders are highly prevalent among healthcare workers (HCWs) globally, with occupational stress (OS) being a major underlying cause. Psychological capital (PsyCap) may serve as a protective resource, yet its combined relationship with OS and sleep quality remains underexplored. **Objectives**: We aimed to identify distinct OS-PsyCap profiles among HCWs, examine their associations with sleep quality, and investigate the mediating and moderating roles of PsyCap. **Methods**: A cross-sectional study was conducted among 1046 HCWs in Shanghai in December 2024. The Job Content Questionnaire, Psychological Capital Questionnaire, and Pittsburgh Sleep Quality Index were used to measure OS, PsyCap, and sleep quality, respectively. Latent profile analysis (LPA) was conducted to identify OS–PsyCap subgroups. Generalized linear models (GLM) and moderation and mediation analyses were performed to examine associations and to elucidate the role of PsyCap. **Results**: HCWs were divided into two distinct profiles: Class 1 (low OS and high PsyCap, 45.2%) and Class 2 (high OS and low PsyCap, 54.8%). HCWs in Class 2 had significantly poorer self-rated health and more sleep disturbances. Higher job demands and organizational support were associated with worse sleep outcomes, while higher autonomy and higher PsyCap were protective. In mediation and moderation analyses, PsyCap mediated 16.4–37.8% of OS–sleep relationships but also amplified OS effects on certain sleep outcomes under high stress. **Conclusions:** High OS with low PsyCap significantly increased sleep disorder risk among HCWs. PsyCap exhibited dual mechanisms, mediating stress effects while exacerbating sleep issues in high-stress contexts. Targeted interventions should address these HCWs in distinct profiles and pathways.

## 1. Introduction

Sleep disorders represent a significant and pervasive occupational health challenge among healthcare workers (HCWs), exhibiting alarmingly high prevalence rates globally [1,2]. Numerous epidemiological studies have reported that physicians, nurses, and other clinical staff experience sleep disturbances, including insomnia, insufficient sleep duration, and poor sleep quality, at frequencies substantially exceeding those observed in the general population [3,4,5]. Previous studies found over half of shift-working HCWs suffered from inadequate sleep, with nurses being one of the most affected groups [6]. In China, approximately 39.2% of HCWs experienced clinically relevant sleep disturbance [7], and recent evidence from Shanghai showed that over 80% of primary HCWs with high occupational stress exhibited impairment in at least one sleep dimension [8]. Together, these data highlight a growing burden of sleep problems in Chinese HCWs, especially in large metropolitan areas such as Shanghai. As one of China’s major medical hubs, the Shanghai’s healthcare system is characterized by high patient volume and widespread rotating shift schedules, with physicians and nurses commonly working long hours and frequent night duties [9,10], which may further exacerbate occupational stress and sleep disruption [11].

This widespread issue extends beyond personal discomfort, posing serious health risks for practitioners. Chronic sleep problems are strongly linked to increased cardiovascular disease, metabolic issues, weakened immunity, and mental health disorders like depression and anxiety [12,13,14]. Critically, impaired sleep among HCWs elevates the risk of medical errors, diagnostic inaccuracies, and procedural mistakes due to reduced cognitive function and vigilance [15,16,17]. These errors not only jeopardize patient safety but are associated with adverse health outcomes, such as increased adverse event rates, delayed care, and potentially preventable morbidity [18]. Such errors carry severe professional, emotional, and legal consequences for doctors. Addressing sleep disturbances in HCWs is therefore essential for both worker well-being and patient safety.

While multiple factors contribute to sleep disturbances in HCWs—including shift work, long hours work, and lifestyle—the profession’s inherent nature highlights occupational stress (OS) as a major underlying cause [19,20]. In practice, OS in healthcare commonly manifests in workplace pressures such as a high patient volume, limited organizational support, frequent night duties, and strained interprofessional relationships; together these create a high-demand and low-control environment. HCWs therefore often face excessive workloads, profound emotional burdens (dealing with suffering and death), constant high-stakes decision-making, and administrative pressures [21,22,23]. This chronic exposure causes significant job strain, involving high demands with limited autonomy and support. Persistent occupational stress is a well-established precursor to psychological distress and physiological dysregulation [24]. Evidence indicated that the hyperarousal state induced by chronic stress directly disrupts the ability to initiate and maintain restorative sleep [25]. Therefore, the inherent stress of HCWs is a major cause of high sleep disorder among staff, requiring further study.

Beyond the significant recognized role of occupational stress, emerging evidence points to the increasing attention paid to psychological capital (PsyCap) as a potential protective factor [26]. Consisting of four core psychological resources—self-efficacy, hope, resilience, and optimism, PsyCap can help individuals protect against resource depletion and cope with demanding work environments, as validated through multiple studies grounded in the Conservation of Resources theory [27,28]. Prior studies have suggested PsyCap could buffer the adverse effects of job stress and be associated with better sleep and well-being [29,30,31]. For instance, resilience has been associated with lower insomnia risk among nurses [32], while optimism predicts better sleep quality in healthy community participants [33]. Collectively, these findings indicate PsyCap may represent a promising intervention target in mitigating the negative impact of occupational stress on sleep. However, most research has treated PsyCap as a single construct, overlooking how its dimensions may differentially interact with specific stressors (e.g., job demand, autonomy) and shape distinct aspects of sleep quality. Moreover, whether PsyCap mainly mediates, moderates, or simultaneously exerts both roles in the stress–sleep pathway is still debated. Meanwhile, sleep disorders and occupational stress are highly prevalent among HCWs in China [8], indicating an urgent need for targeted prevention strategies [34]. Furthermore, the existing work has relied on variable-centered approaches, masking subgroups of HCWs who may experience distinct combinations of high stress and depleted psychological resources, leaving a lack of understanding of naturally occurring subgroups with different OS–PsyCap profiles. A person-centered analysis, such as Latent Profile Analysis (LPA), is essential for identifying naturally occurring OS–PsyCap profiles. This approach may reveal high risk groups that may be hidden in overall averages and may guide the development of precise profile-specific interventions to support a shift from generalized to precision strategies in protecting HCWs’ mental health [35].

Building on the potential of PsyCap as a protective resource, its interplay with occupational stress and sleep quality likely involves complex pathways. Research clarifying these distinct mechanisms—especially whether moderation, mediation, or both operate—remains limited and inconsistent. For example, PsyCap has been found to mediate the relationship between stress and psychological and physical well-being [36], while in a study of seafarers, a high PsyCap was associated with increased sleepiness when worries about accidents were high, suggesting a reverse buffering effect in certain high-stress situations [37]. Crucially, few studies checked both of these effects (moderation and mediation) at the same time—both for overall PsyCap and its parts like hope or resilience—to see how stress affects sleep in different ways. Figuring this out is key to truly understanding how stress, PsyCap, and sleep connect, to design better support programs among HCWs.

Based on previous evidence, we hypothesized the following: (1) HCWs with high OS and low PsyCap would exhibit poorer sleep quality; (2) PsyCap would partially mediate the associations between OS and sleep outcomes; and (3) PsyCap would moderate the stress–sleep relationship, showing different buffering effects under different stress conditions. Accordingly, we aim to identify distinct OS–PsyCap subgroups (latent profiles) among HCWs, further, to examine how these profiles, along with specific dimensions of OS and PsyCap, are associated with multidimensional sleep quality and investigate PsyCap’s dual roles as a moderator, or a mediator, in the relationship between OS and sleep disorders.

## 2. Methods

### 2.1. Study Design and Data Collection

In December 2024, we conducted a cluster sampling method to select 2 institutions randomly from each of the secondary and primary healthcare facilities tiers in Shanghai. indicating a high level of participation and representativeness. The survey was administered online through a secure electronic questionnaire platform (in Chinese, “Wenjuanxing”), and data were stored on servers accessible only to members of the research team, ensuring confidentiality. The full questionnaire consisted of 105 items in total, including a survey of demographic and work-related characteristics, occupational stress, psychological capital, and sleep quality. Eligible participants included physicians, nurses, technicians, and other allied health professionals in the clinical department who were on-duty during the survey period, while administrative staff and interns were excluded. Within the two-week survey period, a total of 1108 questionnaires were collected from a pool of 1628 clinical staff across the four facilities. After excluding 62 invalid responses, 1046 valid questionnaires were retained, yielding an effective response rate of 94.4%. The average completion time for valid responses was approximately 885 s (about 14.8 min). All participants were informed of the study and provided consent. The study was approved by the Ethics Review Committee of the Shanghai Municipal Center for Disease Control and Prevention (Approval No.: 2023-45).

### 2.2. Questionnaires

#### 2.2.1. General Characteristics

Sociodemographic information was collected using self-designed questionnaires, including age, sex, education level, marital status, and monthly income. For work-related characteristics, we surveyed the department, occupation, job title, hospital level, and work year, as well as the weekly work time, daily break time, inter-day rest time, and night shifts per month. In Shanghai, HCWs typically operate under rotating shift systems, with night shifts structured as 8–12 h rotations depending on the departmental workload and staffing levels. Such organizational features are recognized contributors to occupational stress and irregular sleep patterns among HCWs [38,39]. Additionally, information on lifestyle factors—such as drinking, smoking status, and physical exercise—was collected and considered as potential confounders in further analyses [40]. Self-rated health was also assessed and included as a control variable, as it has been shown to be strongly associated with both psychological capital and sleep quality [41].

#### 2.2.2. Psychological Capital

Psychological capital was measured using the Chinese version of the Psychological Capital Questionnaire (PCQ) developed by Luthans et al. [27]. This 24-item scale comprises four dimensions: self-efficacy, hope, resilience, and optimism. Responses were recorded on a 6-point Likert scale (1 = strongly disagree, 2 = disagree, 3 = somewhat disagree, 4 = somewhat agree, 5 = agree, 6 = strongly agree). The mean score of each dimension and four dimensions reflects the specific and overall psychological capital level, with higher scores indicating higher levels of psychological capital. The Cronbach’s alpha coefficient for the PCQ in this study was 0.939.

#### 2.2.3. Occupational Stress

Occupational stress was assessed using the Job Content Questionnaire (JCQ), developed by Karasek [42], and the Chinese (mainland) version is applicable to the Chinese working population [43]. The scale includes three dimensions: job requirements, autonomy (job control), and occupational support. The questionnaire consists of 22 items scored on a 5-point Likert scale (1 = strongly disagree to 5 = strongly agree), in which decision latitude is reverse-scored, i.e., higher scores represent lower levels of occupational stress. Occupational stress was calculated as the ratio of job demands to decision latitude, with higher scores indicating higher levels of occupational stress. A score higher than 1 was considered positive for occupational stress. The overall Cronbach’s alpha coefficient for the JCQ in this study was 0.823.

#### 2.2.4. Sleep Quality

A validated Chinese language version [44] of the Pittsburgh Sleep Quality Index (PSQI) was used to assess sleep quality [45]. The PSQI is a 19-item self-reported measure that assesses seven components, including the subjective sleep quality, daytime dysfunction, sleep latency, duration, efficiency, disturbances, and use of sleep medication. The total PSQI score ranges from 0 to 21, with higher scores indicating poorer sleep quality. A score higher than 7 indicates clinical sleep disturbance. The Cronbach’s alpha coefficient for the PSQI in this study was 0.742.

### 2.3. Statistical Analysis

Latent Profile Analysis (LPA) was first conducted to identify distinct profiles of HCWs based on their patterns of scores across both three dimensions of psychological capital and four dimensions of occupational stress [46]. Models with two to five profiles were tested. Based on the guidelines for fit indices in a previous study [47], the Akaike information criterion (AIC), Bayesian information criterion (BIC), and entropy were used to determine the optimal number of latent subgroups. Lower AIC and BIC values represented a better model fit. Entropy with a value closer to 1 indicated a better separation of the classes [48]. Once the profiles were identified, each profile was named to best describe its characteristics and to differentiate it from other profiles. Subsequently, Chi-square tests or Fisher’s exact tests on demographics, work-related characteristics, living habits, and health status were conducted to examine the differences of HCWs in different latent classes.

Nonlinear relationships between occupational stress, psychological capital, and sleep quality were estimated initially by restricted cubic spline (RCS) models with three knots. Despite of some nonlinearity observed (Figures S1–S3), we conducted a generalized linear model (GLM) to evaluate these associations, since the trend of correlations was approximately linear. Given that psychological capital may play a role in modifying or mediating the association between occupational stress and sleep quality, we conducted analyses in two parts. First, to investigate the potential moderating effect of psychological capital, we stratified the participants by the median score of psychological capital and conducted separate analyses within each stratum. This approach allowed us to examine whether the association between occupational stress and sleep quality varied depending on the level of psychological capital. Second, to explore the potential mediating effect of psychological capital, we constructed mediation models using the Bootstrap method and examined the average causal-mediated effect (ACME; the sleep quality change mediated by psychological capital), as well as indirect effects, as a proportion of the total effect. Causal mediation analysis was performed using a Monte Carlo simulation, with 5000 iterations. This enabled us to assess whether psychological capital and its dimensions explained part of the relationship between occupational stress and sleep quality.

A two-tailed *p* < 0.05 was considered as statistically significant. All statistical analyses were performed using R (version 4.1.2), along with the R packages rms (version 6.8.0), tidyLPA (version 1.1.0), and mediation (version 4.5.0). A two-sided *p*-value < 0.05 was considered statistically significant.

## 3. Results

### 3.1. Characteristics of Participants

As shown in Table 1, the study participants consisted of 201 males (19.2%) and 845 females (80.8%). The majority of participants were aged ≤40 years (69.7%), had a bachelor’s degree or higher (72.2%), and were married and had children (65.1%). For work-related characteristics, physicians (26.6%) and nurses (48.9%) made up the majority. Only a small percentage (29.8%) worked less than or equal to 40 h per week. More than forty percent had more than 60 min of daily break time (40.4%), and the rest period between shifts was between 12 and 24 h (43.2%). Nearly half were required to work night shifts at least once a month. The prevalence of occupational stress and sleep disorders in HCWs were 73.7% (n = 771) and 34.6% (n = 362), respectively.

Significant differences in the characteristics of HCWs were found between the two classes (Table 1). Specifically, compared to Class 2, Class 1 comprised a higher proportion of middle-aged individuals (31–40 years), had a higher monthly income, was more likely to work in auxiliary departments, included fewer physicians, and reported more break time and exercise habits and shorter weekly working hours and fewer frequent monthly night shifts. Of note, Class 2 had a much higher proportion of HCWs reporting a poor health condition (22.2%), occupational stress (82.2%), and sleep disturbance (45.4%) than Class 1 (7.2%, 63.4%, and 21.6%).

### 3.2. Classes Derived from LPA

**Figure 1:**
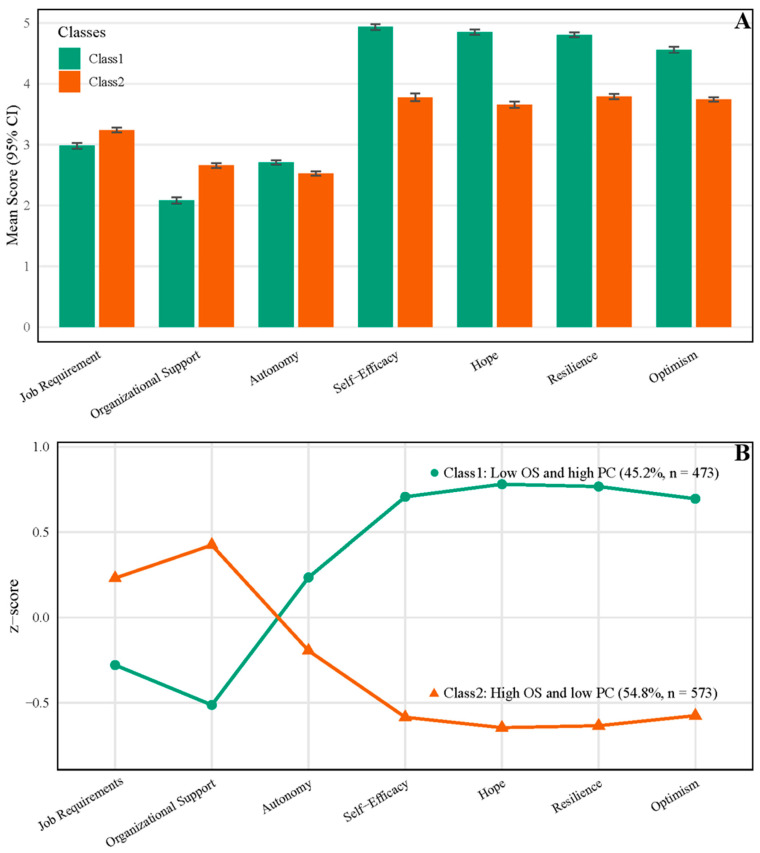
Latent profile analysis results of occupational stress and psychological captivity. (**A**) Comparison of mean scores between Class 1 and Class 2; (**B**) Standardized profiles contrasting Class 1 and Class 2.

Table S1 shows how we chose the “optimal number of latent classes” in our study. Although the model fit indices (AIC/BIC/entropy) suggested that four- or five-class solutions provided a slightly better statistical fit, these models produced very small classes (<10% of the sample), limiting the interpretability and practical value. Given our aim to identify meaningful and distinct profiles, we retained the two-class solution. As shown in Figure 1, Class 1 had a higher proportion of individuals with lower occupational stress and higher psychological capital (n = 473, 45.2%), while Class 2 had a higher proportion of individuals with higher occupational stress and lower psychological capital (n = 573, 54.8%). The mean scores for job requirements, autonomy, occupational support, self-efficacy, hope, resilience, and optimism in Class 1 (Class 2) were 2.98 (3.24), 2.71 (2.53), 2.08 (2.66), 4.93 (3.78), 4.85 (3.66), 4.81 (3.79), and 4.56 (3.74), respectively.

### 3.3. Associations of Sleep Quality with Occupational Stress, Psychological Capital, and Latent Profile

As shown in Table 2, significant associations of occupational stress were observed with different sleep quality dimensions, except for sleep efficiency and the use of sleeping medications. Specifically, job requirements and organizational support were positively associated with the subjective sleep quality (β = 0.21; β = 0.16), daytime dysfunction (β = 0.36, β = 0.25), sleep latency (β = 0.18, β = 0.18), sleep duration (β = 0.22, β = 0.18), and sleep disturbances (β = 0.24; β = 0.17), all with *p*-values < 0.01. Autonomy showed significant negative links with these sleep quality aspects, with β coefficients of 0.20, 0.20, 0.19, 0.18, and 0.19 respectively.

In terms of psychological capital (Table 2), higher levels of all psychological capital dimensions (self-efficacy, hope, resilience, and optimism) and the total score were consistently associated with better subjective sleep quality, less daytime dysfunction, shorter sleep latency, longer sleep duration, fewer sleep disturbances, less use of sleeping medications, and lower PSQI scores (all *p* < 0.001 for most associations).

Compared with Class 1, Class 2 showed significantly worse outcomes across multiple sleep domains: poorer subjective sleep quality, more daytime dysfunction, longer sleep latency, shorter sleep duration, more sleep disturbances, higher use of sleeping medications, and higher global PSQI scores (all *p* ≤ 0.027). The association with poorer sleep efficiency approached significance (β = 0.09, 95% CI: −0.00, 0.19, *p* = 0.063).

### 3.4. The Modifying Effect of Psychological Capital on Occupational Stress–Sleep Quality Associations

As shown in Table 3, in the low psychological capital group, job requirements were consistently positively associated with subjective sleep quality (β = 0.18, 95% CI: 0.04, 0.32), daytime dysfunction (β = 0.33, 95% CI: 0.20, 0.45), sleep duration (β = 0.15, 95% CI: 0.00, 0.29), sleep disturbances (β = 0.29, 95% CI: 0.17, 0.41) and PSQI (β = 1.12, 95% CI: 0.54, 1.70), while organizational support was linked to higher daytime dysfunction (β = 0.20, 95% CI: 0.08, 0.32), shorter sleep duration (β = 0.16, 95% CI: 0.04, 0.29), more sleep disturbances (β = 0.16, 95% CI: 0.05, 0.28) and higher PSQI (β = 0.90, 95% CI: 0.37, 1.42). Notably, autonomy demonstrated protective effects for subjective sleep quality, sleep duration, and reduced sleep disturbances in this group.

Conversely, in the high psychological capital group, job requirements showed broader detrimental effects, significantly impacting all sleep domains except efficiency and medication use. Paradoxically, high psychological capital amplified the negative association between occupational stress and sleep quality—particularly for total occupational stress and daytime dysfunction (β = 0.47 vs. β = 0.25), sleep latency (β = 0.29 vs. β = 0.13), and PSQI (β = 1.55 vs. β = 1.26). Autonomy’s protective role was more pronounced in this group for daytime dysfunction (β = −0.26 vs. β = −0.07) and sleep latency (β = −0.21 vs. β = −0.12). Neither stratum exhibited significant occupational stress-related effects on sleep efficiency. Similar associations were found in stratums of the four dimensions of psychological capital (Tables S2–S5).

### 3.5. The Mediation Effect of Psychological Capital on Occupational Stress–Sleep Quality Associations

Psychological capital and its components significantly mediated the relationships between occupational stress and sleep quality (Tables S6–S10). Self-efficacy, hope, resilience, optimism, and the total score of psychological capital each partially explained how occupational stress influenced sleep outcomes, with the strength of the mediation varying across stress dimensions and sleep domains. Optimism consistently demonstrated the strongest mediating effects, particularly for the impact of organizational support on subjective sleep quality (60.6%, *p* < 0.001) and sleep latency (52.9%, *p* < 0.001). Job requirements were primarily mediated through hope and optimism across most sleep components, while the protective effects of autonomy were substantially explained by all psychological capital dimensions. The mediation effects were weakest for sleep efficiency. Notably, organizational support showed the highest mediation proportions through the total score of psychological capital, especially for subjective sleep quality (64.5%, *p* < 0.001). Total psychological capital mediated 16.4–37.8% of total occupational stress effects on sleep domains. Crucially, all significant mediated pathways demonstrated consistent directional effects: job requirements and organizational support increased sleep impairment through reduced psychological capital, while autonomy improved sleep via enhanced psychological capital.

## 4. Discussion

In this study, two distinct latent profiles were identified among HCWs: Class 2 (low OS and high PsyCap, 45.2%) exhibited significantly poorer sleep quality than Class 1 (high OS and low PsyCap, 54.8%). Multidimensional analysis revealed that higher job requirements and organizational support consistently predicted worse sleep quality, whereas higher autonomy and higher PsyCap levels were of protective effects. PsyCap demonstrated dual mechanistic roles—as a significant mediator explaining stress–sleep pathways and as a potential vulnerability amplifier under high stress exposure.

The observed prevalence of occupational stress (73.7%) and sleep disturbance (34.6%) among Shanghai HCWs aligned with previous reports highlighting these issues as endemic within the profession [9], though slightly exceeding some pre-pandemic benchmarks [7]. Similarly, the identified Class 1 (45.2%) and Class 2 (54.8%) profiles resonating with the emerging evidence indicated co-occurring resource deficits and job strain are common [21]. Critically, the Class 2 represented a vulnerable cohort requiring urgent intervention, exhibiting dramatically elevated rates of poor self-rated health (22.2% vs. 7.2%), occupational stress (82.2% vs. 63.4%), and sleep disturbance (45.4% vs. 21.6%) compared to their resilient counterparts. Profile membership was strongly linked to modifiable work conditions: the vulnerable profile was characterized by a lower income, shorter work break time, longer weekly hours, and more frequent night shifts—factors directly contributing to Karasek’s job strain model (high requirements/high support/low autonomy) [42]. This underscored organizational support as a key driver of adverse stress-resource configurations [49,50]. The LPA approach proved superior to traditional variable-centered methods by revealing how occupational stress dimensions and PsyCap components naturally interact within individuals, capturing real-world heterogeneity [49]. Traditional analyses (e.g., regressions) have often obscured these synergistic patterns, potentially overlooking high-risk subgroups like the one identified here, who faced compounded risk due to concurrent high stress exposure and low psychological resources. This person-centered insight is vital for designing tailored profile-specific interventions rather than one-size-fits-all approaches.

Our findings robustly confirmed the known relationships between occupational psychosocial factors and sleep quality in HCWs. The detrimental associations of higher job requirements and lower autonomy with poorer subjective sleep quality, longer sleep latency, shorter sleep duration, increased sleep disturbances, and higher daytime dysfunction aligned consistently with the Job Demand–Resources (JD-R) model and prior studies [51,52]. Excessive demands may lead to insufficient sleep due to an excessive workload and may also cause persistent excitement, making it difficult to fall asleep or resulting in shallow sleep [53]. Job stressors, including role conflict and repetitive tasks, are directly linked to sleep disorders, independent of working hours and lifestyle [20,54]. Notably, while organizational support generally correlated with improved sleep [55], some studies suggest counterintuitive findings in high-stress contexts consistent with our findings. One study showed that increased organizational support was associated with decreased insomnia symptoms among frontline staff [10]. This paradox, coupled with the LPA results showing organizational support co-occurring with high job requirements and inversely with autonomy in the vulnerable profile, suggested contextual complexity [56]. In high-strain healthcare environments, organizational support may paradoxically reflect or accompany intensified workloads (e.g., supportive resources provided because demands are overwhelming) or signify the inadequate quality of the support, failing to offset demands [51]. In addition, psychological capital functions as a robust personal resource, in which all components were negatively correlated with sleep disorders. The significantly worse sleep in the class with high OS and low PsyCap profile also underscored the synergistic detriment of combined job strain and depleted psychological resources [57]. The lack of significant associations for sleep efficiency and medication use in many models may reflect methodological limitations: self-reported efficiency estimates are often inaccurate versus actigraphy [58], while medication use was comparatively rare in our sample (≤10%).

Two key functions of PsyCap in HCWs have been revealed in our study. First, the total PsyCap significantly mediated the occupational stress–sleep quality relationship, explaining 16.4–37.8% of effects. Optimism was the strongest mediator, particularly for the impact of organizational support on subjective sleep quality (mediating 60.6%). Job requirements mainly affected sleep through reduced hope and optimism, while autonomy improved sleep by boosting all PsyCap components. The Conservation of Resources theory supports our findings, where autonomy fosters psychological resource accumulation. For example, PsyCap was found to enhance job embeddedness and employee retention in the hospitality industry [59]. Second, we observed a paradoxical moderation effect. High PsyCap unexpectedly amplified the association between the total occupational stress and certain sleep problems. For example, the association of occupational stress with daytime dysfunction was stronger in high-PsyCap workers (β = 0.47) than in low-PsyCap workers (β = 0.25). Valdersnes et al.’s study showed similar results among seafarers, where high PsyCap was associated with increased sleepiness when worry about accidents was high [37]. These findings suggested that HCWs with a higher PsyCap may perceive and react more strongly to job stressors. Rather than indicating a vulnerability, this heightened sensitivity implies that reducing external job demands or improving work autonomy could yield higher benefits for their sleep quality. The dual roles of PsyCap therefore have practical implications: (1) for low-PsyCap staff, cultivating optimism could disrupt stress–sleep pathways; (2) for high-PsyCap workers, reducing uncontrollable job demands may be critical; (3) workplace interventions that enhance autonomy can further reinforce PsyCap. The consistent mediation pathways confirmed PsyCap’s role as a conduit for stress effects, while its context-dependent moderation highlights the need for tailored specific intervention strategies in healthcare groups.

Several strengths in this study are worth noting, including a relatively large sample size, the application of LPA to identify naturally occurring occupational stress–PsyCap subgroups among HCWs, and a comprehensive multidimensional assessment of associations of sleep quality with occupational stress and psychological capital. Critically, we simultaneously examined both moderation and mediation mechanisms—addressing a key gap in understanding psychological capital’s dual roles. However, this study has some limitations. The cross-sectional design precluded causal inferences about the observed stress–PsyCap–sleep pathways. Self-reported measures may introduce recall or social desirability bias, particularly for sensitive outcomes like sleep quality. Our Shanghai-based sample limits the generalizability of results to other healthcare systems and/or cultural contexts in other regions. Additionally, the generalizability of our findings may be limited by the sampling timeframe, as confinement to the month of December could amplify associations specific to the high-demand winter months. While the two-class LPA solution was clinically interpretable, higher-class models warrant exploration in future studies. Finally, despite adjusting for key confounders, residual confounding from unmeasured factors (e.g., work environment specifics) remains possible.

## 5. Conclusions

In summary, this study identified a vulnerable high-stress and low psychological capital profile among healthcare workers, demonstrating significantly elevated sleep disturbance risk. Psychological capital’s dual role was confirmed in that it strongly mediated stress–sleep pathways (particularly through optimism), yet paradoxically amplified occupational stress effects on sleep disorder in resilient individuals. These findings underscored that sleep interventions among health workers must be profile-specific. Future longitudinal studies should verify causal pathways and test tailored interventions addressing these distinct mechanisms.

## Figures and Tables

**Table 1:** Characteristics of study population (N = 1046).

Variable	Total	Class 1 ^a^	Class 2 ^b^	*p*-Value ^c^
**Demographic characteristics**				
Age (years)				**0.038**
≤30	395 (37.8%)	162 (34.2%)	233 (40.7%)	
31–40	334 (31.9%)	162 (34.2%)	172 (30.0%)	
41–50	225 (21.5%)	98 (20.7%)	127 (22.2%)	
>50	92 (8.8%)	51 (10.8%)	41 (7.2%)	
Sex				0.826
female	845 (80.8%)	384 (81.2%)	461 (80.5%)	
male	201 (19.2%)	89 (18.8%)	112 (19.5%)	
Education level				0.148
junior college and lower	291 (27.8%)	145 (30.7%)	146 (25.5%)	
bachelor’s degree	685 (65.5%)	300 (63.4%)	385 (67.2%)	
master’s degree and higher	70 (6.7%)	28 (5.9%)	42 (7.3%)	
Marital status				0.187
unmarried	253 (24.2%)	107 (22.6%)	146 (25.5%)	
married without child/ren	95 (9.1%)	43 (9.1%)	52 (9.1%)	
married with child/ren	681 (65.1%)	319 (67.4%)	362 (63.2%)	
other	17 (1.6%)	4 (0.8%)	13 (2.3%)	
Monthly income (CNY)				**0.025**
≤1 w	590 (56.4%)	265 (56.0%)	325 (56.7%)	
1–1.5 w	400 (38.2%)	173 (36.6%)	227 (39.6%)	
>1.5 w	56 (5.4%)	35 (7.4%)	21 (3.7%)	
**Work-related characteristics**				
Department				
clinic	617 (59.0%)	252 (53.3%)	365 (63.7%)	**<0.001**
technology	112 (10.7%)	49 (10.4%)	63 (11.0%)	
auxiliary	317 (30.3%)	172 (36.4%)	145 (25.3%)	
Occupation				**0.005**
nurse	512 (48.9%)	245 (51.8%)	267 (46.6%)	
physician	278 (26.6%)	103 (21.8%)	175 (30.5%)	
technician	70 (6.7%)	29 (6.1%)	41 (7.2%)	
apothecary	82 (7.8%)	37 (7.8%)	45 (7.9%)	
others	104 (9.9%)	59 (12.5%)	45 (7.9%)	
Job title				0.339
primary	577 (55.2%)	253 (53.5%)	324 (56.5%)	
intermediate	397 (38.0%)	182 (38.5%)	215 (37.5%)	
senior	72 (6.9%)	38 (8.0%)	34 (5.9%)	
Hospital level				0.868
primary level	167 (16.0%)	77 (16.3%)	90 (15.7%)	
secondary level	879 (84.0%)	396 (83.7%)	483 (84.3%)	
Work year (year)				0.088
<5	266 (25.4%)	117 (24.7%)	149 (26.0%)	
5–10	264 (25.2%)	104 (22.0%)	160 (27.9%)	
10–20	248 (23.7%)	122 (25.8%)	126 (22.0%)	
>20	268 (25.6%)	130 (27.5%)	138 (24.1%)	
Weekly worktime (hour)				**0.031**
≤40	312 (29.8%)	147 (31.1%)	165 (28.8%)	
41–48	572 (54.7%)	270 (57.1%)	302 (52.7%)	
49–54	101 (9.7%)	34 (7.2%)	67 (11.7%)	
≥55	61 (5.8%)	22 (4.7%)	39 (6.8%)	
Daily break time (min)				**0.003**
no	59 (5.6%)	16 (3.4%)	43 (7.5%)	
10–30	228 (21.8%)	91 (19.2%)	137 (23.9%)	
30–60	336 (32.1%)	156 (33.0%)	180 (31.4%)	
>60	423 (40.4%)	210 (44.4%)	213 (37.2%)	
Inter-day rest time (hour)				0.072
<8	118 (11.3%)	53 (11.2%)	65 (11.3%)	
8–12	418 (40.0%)	172 (36.4%)	246 (42.9%)	
12–24	452 (43.2%)	215 (45.5%)	237 (41.4%)	
>24	58 (5.5%)	33 (7.0%)	25 (4.4%)	
Night shifts per month				**<0.001**
none	528 (50.5%)	273 (57.7%)	255 (44.5%)	
1–3	165 (15.8%)	75 (15.9%)	90 (15.7%)	
4–7	318 (30.4%)	112 (23.7%)	206 (36.0%)	
>8	35 (3.3%)	13 (2.7%)	22 (3.8%)	
**Lifestyle factors**				
Drinking				0.166
no	975 (93.2%)	447 (94.5%)	528 (92.1%)	
yes	71 (6.8%)	26 (5.5%)	45 (7.9%)	
Smoking				0.849
no	1006 (96.2%)	456 (96.4%)	550 (96.0%)	
yes	40 (3.8%)	17 (3.6%)	23 (4.0%)	
Physical exercise				**0.026**
no	804 (76.9%)	348 (73.6%)	456 (79.6%)	
yes	242 (23.1%)	125 (26.4%)	117 (20.4%)	
**Health status**				
Self-health evaluation				**<0.001**
good	239 (22.8%)	168 (35.5%)	71 (12.4%)	
general	646 (61.8%)	271 (57.3%)	375 (65.4%)	
poor	161 (15.4%)	34 (7.2%)	127 (22.2%)	
Occupational stress				**<0.001**
no	275 (26.3%)	173 (36.6%)	102 (17.8%)	
yes	771 (73.7%)	300 (63.4%)	471 (82.2%)	
Sleep disturbance				**<0.001**
no	684 (65.4%)	371 (78.4%)	313 (54.6%)	
yes	362 (34.6%)	102 (21.6%)	260 (45.4%)	

Note: ^a^ Class 1 included people with low occupational stress and high psychological capital; ^b^ Class 2 included people with high occupational stress and low psychological capital; ^c^ Bold consent means *p* < 0.05.

**Table 2:** Associations of sleep quality with occupational stress, psychological capital, and latent profile (N = 1046).

Sleep Quality	Subjective Sleep Quality		Daytime Dysfunction		Sleep Latency		Sleep Duration	
	β (95%CI)	*p* Value	β (95%CI)	*p* Value	β (95%CI)	*p* Value	β (95%CI)	*p* Value
**Occupational stress**								
Job requirements	0.21 (0.11, 0.30)	**<0.001**	0.36 (0.27, 0.45)	**<0.001**	0.18 (0.06, 0.31)	**0.004**	0.22 (0.12, 0.31)	**<0.001**
Organizational support	0.16 (0.08, 0.24)	**<0.001**	0.25 (0.18, 0.33)	**<0.001**	0.18 (0.08, 0.29)	**<0.001**	0.18 (0.10, 0.26)	**<0.001**
Autonomy	−0.20 (−0.30, −0.09)	**<0.001**	−0.20 (−0.30, −0.10)	**<0.001**	−0.19 (−0.33, −0.05)	**0.007**	−0.18 (−0.28, −0.07)	**0.001**
Total score	0.29 (0.17, 0.40)	**<0.001**	0.39 (0.28, 0.50)	**<0.001**	0.21 (0.06, 0.37)	**0.007**	0.25 (0.13, 0.37)	**<0.001**
**Psychological capital**								
Self-efficacy	−0.15 (−0.20, −0.10)	**<0.001**	−0.18 (−0.23, −0.13)	**<0.001**	−0.16 (−0.23, −0.09)	**<0.001**	−0.08 (−0.14, −0.03)	**0.002**
Hope	−0.19 (−0.25, −0.14)	**<0.001**	−0.23 (−0.28, −0.18)	**<0.001**	−0.18 (−0.26, −0.11)	**<0.001**	−0.11 (−0.17, −0.05)	**<0.001**
Resilience	−0.19 (−0.25, −0.13)	**<0.001**	−0.25 (−0.31, −0.20)	**<0.001**	−0.16 (−0.25, −0.08)	**<0.001**	−0.10 (−0.16, −0.03)	**0.004**
Optimism	−0.24 (−0.31, −0.17)	**<0.001**	−0.27 (−0.33, −0.20)	**<0.001**	−0.25 (−0.34, −0.15)	**<0.001**	−0.16 (−0.23, −0.08)	**<0.001**
Total score	−0.26 (−0.33, −0.19)	**<0.001**	−0.31 (−0.38, −0.25)	**<0.001**	−0.25 (−0.34, −0.16)	**<0.001**	−0.15 (−0.22, −0.07)	**<0.001**
**Latent profile (Refer to Class 1)**								
Class 2	0.26 (0.17, 0.35)	**<0.001**	0.34 (0.26, 0.43)	**<0.001**	0.28 (0.16, 0.40)	**<0.001**	0.17 (0.07, 0.26)	**0.001**
**Sleep Quality**	**Sleep Efficiency**		**Sleep Disturbances**		**Use of Sleeping Medications**		**PSQI**	
	**β (95%CI)**	***p* Value**	**β (95%CI)**	***p* Value**	**β (95%CI)**	***p* Value**	**β (95%CI)**	***p* Value**
**Occupational stress**								
Job requirements	0.03 (−0.07, 0.13)	0.573	0.24 (0.16, 0.32)	**<0.001**	0.04 (−0.04, 0.11)	0.307	1.28 (0.89, 1.66)	**<0.001**
Organizational support	0.06 (−0.02, 0.15)	0.136	0.17 (0.10, 0.23)	**<0.001**	0.04 (−0.02, 0.10)	0.206	1.05 (0.73, 1.37)	**<0.001**
Autonomy	−0.05 (−0.16, 0.06)	0.404	−0.19 (−0.28, −0.10)	**<0.001**	0.05 (−0.04, 0.13)	0.263	−0.96 (−1.39, −0.52)	**<0.001**
Total score	0.07 (−0.05, 0.20)	0.260	0.31 (0.21, 0.41)	**<0.001**	0.02 (−0.08, 0.11)	0.744	1.55 (1.06, 2.03)	**<0.001**
**Psychological capital**								
Self-efficacy	−0.06 (−0.12, −0.01)	**0.026**	−0.11 (−0.15, −0.07)	**<0.001**	−0.06 (−0.10, −0.02)	**0.006**	−0.80 (−1.02, −0.59)	**<0.001**
Hope	−0.04 (−0.10, 0.02)	0.177	−0.13 (−0.18, −0.08)	**<0.001**	−0.04 (−0.08, 0.00)	0.074	−0.93 (−1.15, −0.70)	**<0.001**
Resilience	−0.04 (−0.11, 0.02)	0.195	−0.14 (−0.20, −0.09)	**<0.001**	−0.07 (−0.12, −0.02)	**0.005**	−0.96 (−1.22, −0.70)	**<0.001**
Optimism	−0.08 (−0.16, −0.00)	**0.040**	−0.15 (−0.21, −0.08)	**<0.001**	−0.06 (−0.12, −0.00)	**0.034**	−1.20 (−1.49, −0.90)	**<0.001**
Total score	−0.08 (−0.15, −0.00)	**0.041**	−0.18 (−0.24, −0.12)	**<0.001**	−0.08 (−0.13, −0.02)	**0.005**	−1.31 (−1.59, −1.02)	**<0.001**
**Latent profile (Refer to Class 1)**								
Class 2	0.09 (−0.00, 0.19)	0.063	0.21 (0.13, 0.28)	**<0.001**	0.08 (0.01, 0.15)	**0.027**	1.43 (1.06, 1.81)	**<0.001**

Note: Models were adjusted according to sex, age, occupation, monthly income, night shifts per month, daily work break time, exercise habits, weekly worktime, and self-health evaluation. Bold consent means *p* < 0.05.

**Table 3:** Associations of occupational stress with sleep quality in low and high psychological capital classes.

Total Score of Psychological Capital	Subjective Sleep Quality		Daytime Dysfunction		Sleep Latency		Sleep Duration	
	β (95%CI)	*p* Value	β (95%CI)	*p* Value	β (95%CI)	*p* Value	β (95%CI)	*p* Value
**Below median**								
Job requirements	0.18 (0.04, 0.32)	**0.011**	0.33 (0.20, 0.45)	**<0.001**	0.11 (−0.08, 0.30)	0.247	0.15 (0.00, 0.29)	**0.043**
Organizational support	0.12 (−0.01, 0.24)	0.069	0.20 (0.08, 0.32)	**0.001**	0.09 (−0.07, 0.26)	0.279	0.16 (0.04, 0.29)	**0.012**
Autonomy	−0.18 (−0.33, −0.03)	**0.022**	−0.07 (−0.22, 0.07)	0.325	−0.12 (−0.33, 0.08)	0.235	−0.19 (−0.34, −0.03)	**0.019**
Total score	0.24 (0.09, 0.40)	**0.002**	0.25 (0.11, 0.40)	**0.001**	0.13 (−0.07, 0.34)	0.210	0.20 (0.04, 0.35)	**0.016**
**Equal or above median**								
Job requirements	0.20 (0.06, 0.33)	**0.004**	0.30 (0.18, 0.42)	**<0.001**	0.20 (0.04, 0.37)	**0.017**	0.25 (0.11, 0.39)	**<0.001**
Organizational support	0.09 (−0.02, 0.20)	0.110	0.16 (0.06, 0.26)	**0.002**	0.13 (−0.01, 0.27)	0.064	0.16 (0.04, 0.27)	**0.007**
Autonomy	−0.16 (−0.31, −0.01)	**0.032**	−0.26 (−0.39, −0.12)	**<0.001**	−0.21 (−0.40, −0.03)	**0.026**	−0.13 (−0.28, 0.03)	0.104
Total score	0.29 (0.09, 0.48)	**0.004**	0.47 (0.29, 0.64)	**<0.001**	0.29 (0.05, 0.53)	**0.019**	0.30 (0.10, 0.50)	**0.003**
	**Sleep Efficiency**		**Sleep Disturbances**		**Use of sleeping Medications**		**PSQI**	
	**β (95%CI)**	***p* Value**	**β (95%CI)**	***p* Value**	**β (95%CI)**	***p* Value**	**β (95%CI)**	***p* Value**
**Below median**								
Job requirements	0.07 (−0.09, 0.23)	0.398	0.29 (0.17, 0.41)	**<0.001**	−0.00 (−0.12, 0.12)	0.999	1.12 (0.54, 1.70)	**<0.001**
Organizational support	0.10 (−0.04, 0.24)	0.177	0.16 (0.05, 0.28)	**0.004**	0.06 (−0.05, 0.18)	0.269	0.90 (0.37, 1.42)	**0.001**
Autonomy	−0.10 (−0.27, 0.07)	0.253	−0.22 (−0.35, −0.08)	**0.002**	0.07 (−0.07, 0.20)	0.332	−0.81 (−1.45, −0.17)	**0.014**
Total score	0.10 (−0.08, 0.27)	0.270	0.33 (0.20, 0.47)	**<0.001**	0.01 (−0.13, 0.15)	0.929	1.26 (0.61, 1.91)	**<0.001**
**Equal or above median**								
Job requirements	−0.05 (−0.18, 0.08)	0.424	0.15 (0.05, 0.26)	**0.005**	0.05 (−0.04, 0.14)	0.242	1.10 (0.59, 1.62)	**<0.001**
Organizational support	0.02 (−0.09, 0.13)	0.748	0.09 (−0.00, 0.18)	0.057	−0.02 (−0.09, 0.05)	0.629	0.63 (0.19, 1.07)	**0.005**
Autonomy	0.01 (−0.14, 0.15)	0.904	−0.13 (−0.25, −0.01)	**0.031**	0.06 (−0.04, 0.15)	0.251	−0.83 (−1.41, −0.24)	**0.006**
Total score	−0.01 (−0.20, 0.18)	0.937	0.23 (0.08, 0.39)	**0.004**	−0.02 (−0.15, 0.11)	0.746	1.55 (0.79, 2.30)	**<0.001**

Note: Models were adjusted with sex, age, occupation, monthly income, night-shift per month, daily break time, exercise habits, weekly worktime and self-health evaluation. Bold consent means *p* < 0.05.

## Data Availability

The data presented in this study are available on request from the corresponding author. The data are not publicly available due to privacy/ethical reasons.

## Supplementary Materials

The following supporting information can be downloaded at https://www.mdpi.com/article/10.3390/healthcare13233109/s1, Figure S1: The relationships of occupational stress with sleep quality fitted with restricted cubic spline models. Figure S2: The relationships of psychological capital fitted with sleep quality restricted cubic spline models. Figure S3: The relationships of occupational stress with psychological capital fitted with restricted cubic spline models. Table S1: Goodness of fit measures for the two–five latent profile analysis. Table S2: Associations of occupational stress with sleep quality in low and high self-efficacy classes. Table S3: Associations of occupational stress with sleep quality in low and high hope classes. Table S4: Associations of occupational stress with sleep quality in low and high resilience classes. Table S5: Associations of occupational stress with sleep quality in low and high optimism classes. Table S6: Results of mediation analysis of self-efficacy. Table S7: Results of mediation analysis of hope. Table S8: Results of mediation analysis of resilience. Table S9: Results of mediation analysis of optimism. Table S10: Results of mediation analysis of the total score of PsyCap.
