# Supplementary material for: Latent Profile Analysis of Occupational Stress and Psychological Capital and the Dual Mechanisms of Psychological Capital in Healthcare Workers’ Sleep Quality
Source: Healthcare (Basel). 2025 Nov 28;13(23):3109. doi: 10.3390/healthcare13233109 (PMC12692588; doi:10.3390/healthcare13233109)
Supplement: Supplementary file 1 [file healthcare-13-03109-s001.zip › healthcare-3972210-supplementary.pdf]

Supplement materials

Figure S1 The relationships of occupational stress with sleep quality fitted with restricted cubic spline models.

Figure S2 The relationships of psychological capital with fitted with sleep quality restricted cubic spline models.

Figure S3 The relationships of occupational stress with psychological capital fitted with restricted cubic spline models.

Table S1 Goodness of fit measures for the 2-5 latent profile analysis.

Table S2 Associations of occupational stress with sleep quality in low and high self-efficacy classes.

Table S3 Associations of occupational stress with sleep quality in low and high hope classes.

Table S4 Associations of occupational stress with sleep quality in low and high resilience classes.

Table S5 Associations of occupational stress with sleep quality in low and high optimism classes.

Table S6 Results of mediation analysis of self-efficacy.

Table S7 Results of mediation analysis of hope.

Table S8 Results of mediation analysis of resilience.

Table S9 Results of mediation analysis of optimism.

Table S10 Results of mediation analysis of total score of PsyCap.

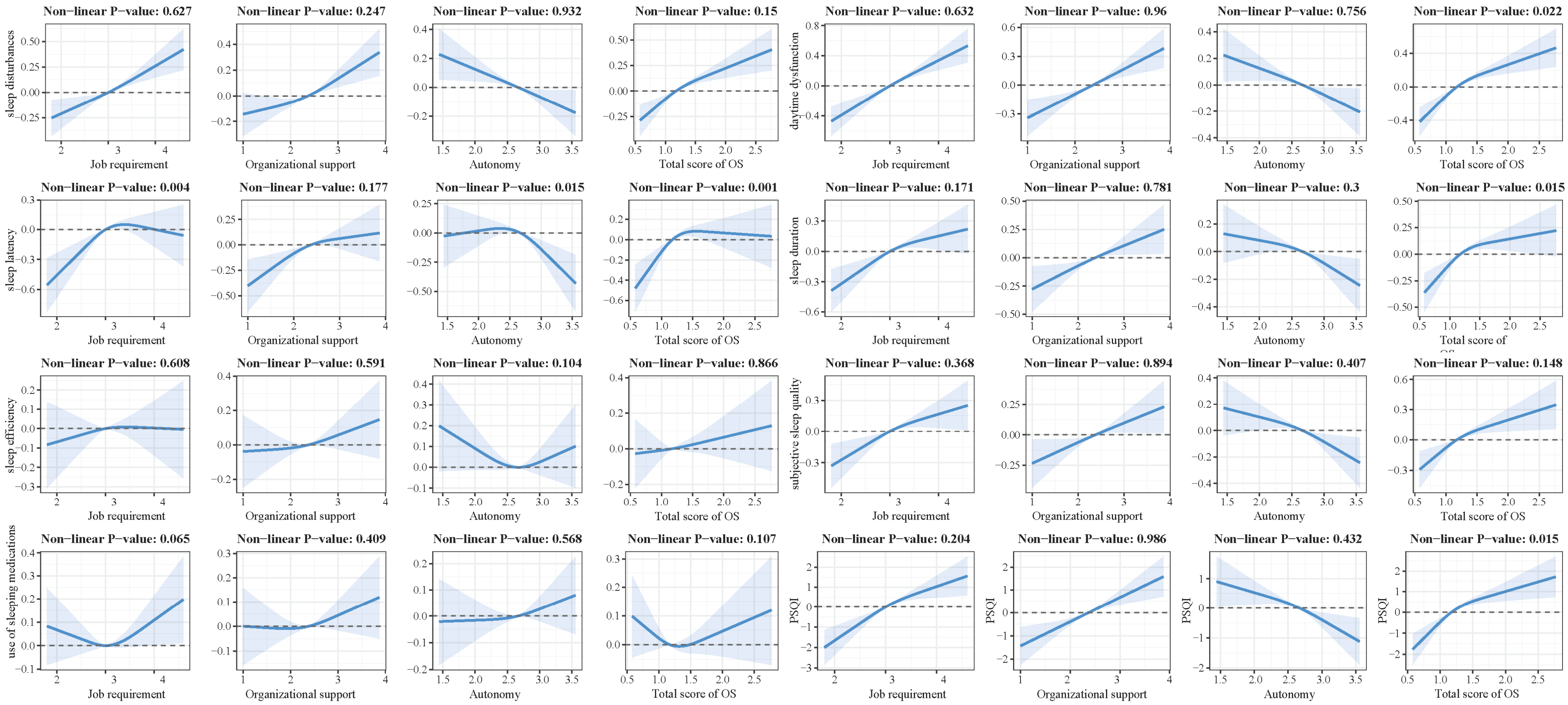

Figure S1 The relationships of occupational stress with sleep quality fitted with restricted cubic spline models. Models were adjusted with sex, age, occupation, monthly income, night-shift per month, intra-day rest time, exercise habits, weekly worktime and self-health evaluation.

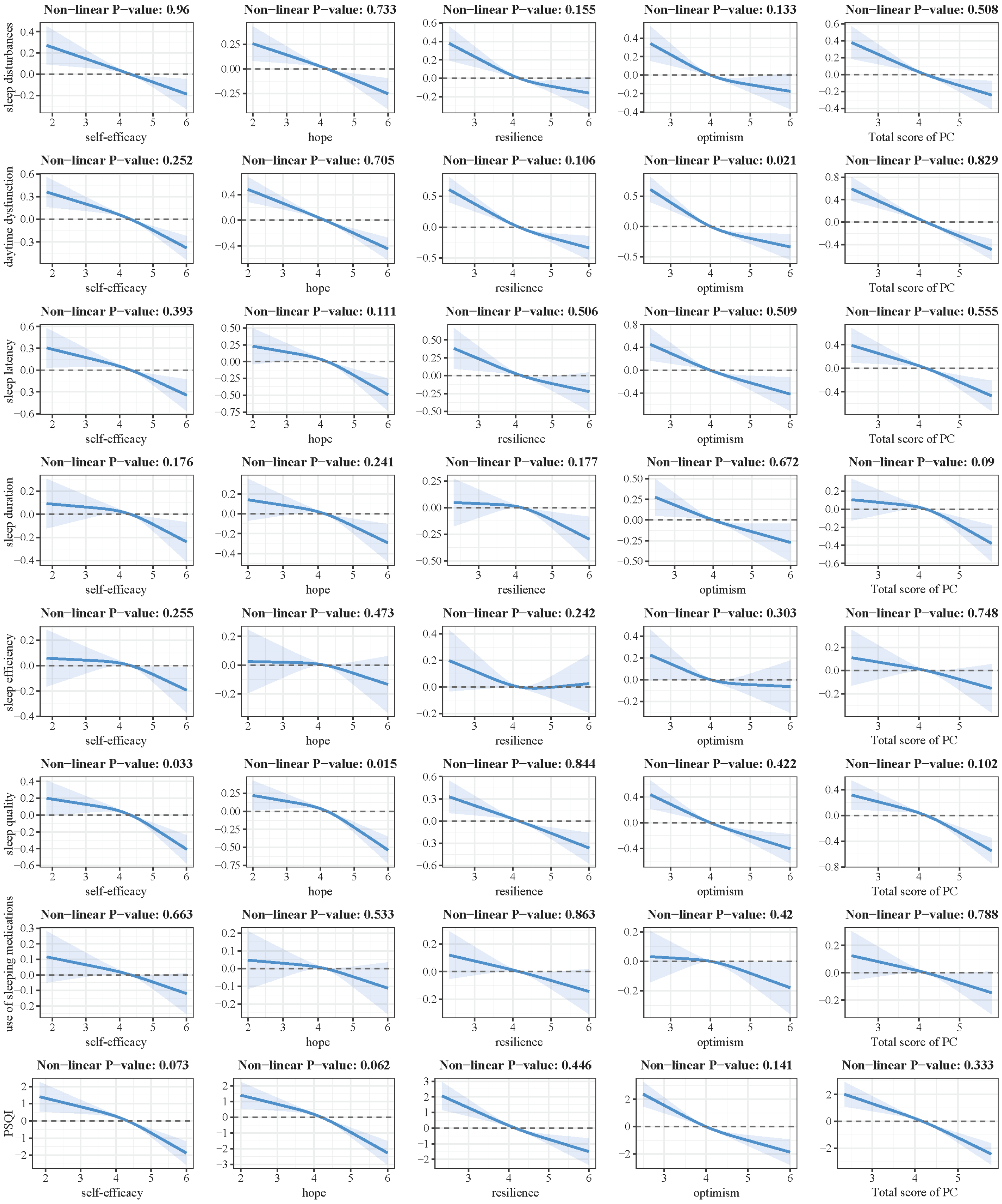

Figure S2 The relationships of psychological capital with fitted with sleep quality restricted cubic spline models. Models were adjusted with sex, age, occupation, monthly income, night-shift per month, intra-day rest time, exercise habits, weekly worktime and self-health evaluation.

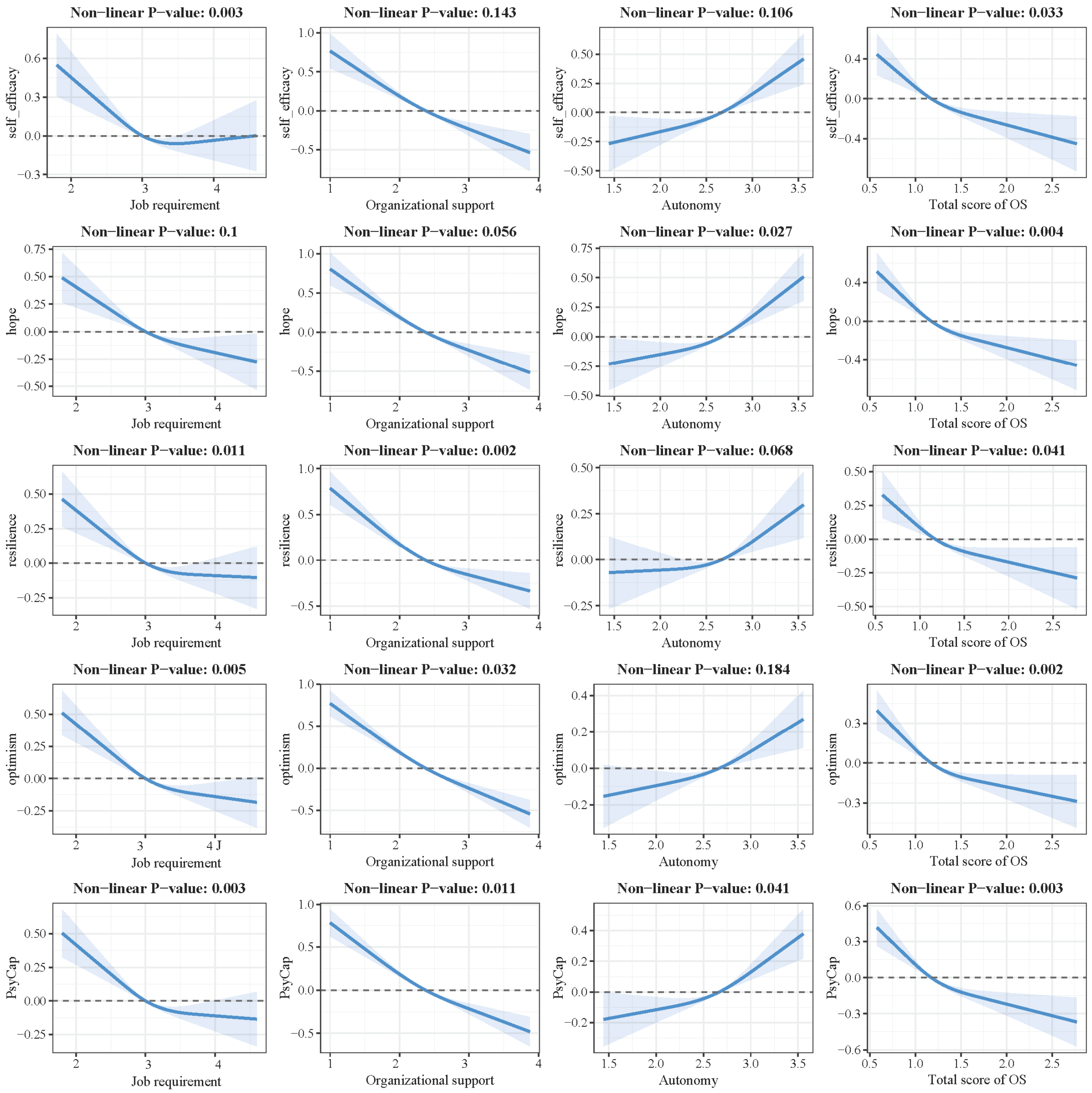

Figure S3 The relationships of occupational stress with psychological capital fitted with restricted cubic spline models. Models were adjusted with sex, age, occupation, monthly income, night-shift per month, intra-day rest time, exercise habits, weekly worktime and self-health evaluation.

Table S1 Goodness of fit measures for the 2-5 latent profile analysis.

Classes	AIC	BIC	Entropy	Class 1%	Class 2%	Class 3%	Class 4%	Class 5%
2	19127.69	19235.65	0.842	45.2	54.8			
3	18545.12	18693.70	0.875	39.8	7.2	53.0		
4	18018.82	18207.02	**0.904**	39.3	6.4	6.2	48.1	
5	**17930.36**	**18158.19**	0.881	39.5	6.0	5.8	44.0	4.7

Table S2 Associations of occupational stress with sleep quality in low and high self-efficacy classes.

**Self-efficacy**	Subjective sleep quality		Daytime dysfunction		Sleep latency		Sleep duration	
	β (95%CI)	*p* value	β (95%CI)	*p* value	β (95%CI)	*p* value	β (95%CI)	*p* value
**Below median (4.33)**								
Job requirement	0.28 (0.15, 0.41)	**<0.001**	0.41 (0.28, 0.53)	**<0.001**	0.20 (0.02, 0.39)	**0.033**	0.16 (0.02, 0.30)	**0.026**
Organizational support	0.11 (-0.01, 0.23)	0.064	0.23 (0.12, 0.35)	**<0.001**	0.13 (-0.04, 0.29)	0.133	0.17 (0.04, 0.29)	**0.009**
Autonomy	-0.14 (-0.29, 0.01)	0.070	-0.13 (-0.28, 0.01)	0.077	-0.08 (-0.29, 0.13)	0.456	-0.20 (-0.36, -0.05)	**0.011**
Total score	0.27 (0.12, 0.42)	**<0.001**	0.33 (0.19, 0.48)	**<0.001**	0.15 (-0.06, 0.36)	0.162	0.22 (0.06, 0.37)	**0.007**
**Equal or above median**								
Job requirement	0.12 (-0.02, 0.25)	0.085	0.29 (0.17, 0.42)	**<0.001**	0.14 (-0.03, 0.30)	0.101	0.25 (0.12, 0.39)	**<0.001**
Organizational support	0.14 (0.03, 0.25)	**0.013**	0.19 (0.09, 0.29)	**<0.001**	0.14 (0.00, 0.27)	**0.044**	0.17 (0.06, 0.28)	**0.003**
Autonomy	-0.19 (-0.34, -0.05)	**0.010**	-0.22 (-0.36, -0.08)	**0.003**	-0.23 (-0.41, -0.05)	**0.013**	-0.15 (-0.30, 0.01)	0.059
Total score	0.25 (0.06, 0.44)	**0.011**	0.42 (0.25, 0.60)	**<0.001**	0.24 (0.01, 0.48)	**0.043**	0.30 (0.10, 0.50)	**0.003**
	Sleep efficiency		Sleep disturbances		Use of sleeping medications		PSQI	
	β (95%CI)	*p* value	β (95%CI)	*p* value	β (95%CI)	*p* value	β (95%CI)	*p* value
**Below median**								
Job requirement	0.10 (-0.06, 0.27)	0.221	0.33 (0.21, 0.45)	**<0.001**	-0.00 (-0.12, 0.12)	0.992	1.48 (0.92, 2.05)	**<0.001**
Organizational support	0.03 (-0.12, 0.18)	0.709	0.18 (0.07, 0.29)	**0.002**	0.05 (-0.06, 0.16)	0.373	0.90 (0.38, 1.41)	**0.001**
Autonomy	-0.06 (-0.24, 0.12)	0.527	-0.26 (-0.40, -0.13)	**<0.001**	0.13 (-0.00, 0.26)	0.056	-0.75 (-1.39, -0.11)	**0.023**
Total score	0.09 (-0.09, 0.28)	0.312	0.39 (0.25, 0.52)	**<0.001**	-0.03 (-0.16, 0.11)	0.669	1.42 (0.79, 2.05)	**<0.001**
**Equal or above median**								
Job requirement	-0.05 (-0.17, 0.07)	0.415	0.14 (0.04, 0.25)	**0.008**	0.08 (-0.01, 0.17)	0.100	0.97 (0.46, 1.48)	**<0.001**
Organizational support	0.05 (-0.05, 0.15)	0.306	0.12 (0.04, 0.21)	**0.006**	0.01 (-0.06, 0.09)	0.737	0.82 (0.40, 1.24)	**<0.001**
Autonomy	-0.02 (-0.15, 0.12)	0.818	-0.11 (-0.23, 0.01)	0.078	-0.00 (-0.11, 0.10)	0.948	-0.92 (-1.50, -0.34)	**0.002**
Total score	0.01 (-0.16, 0.18)	0.924	0.19 (0.03, 0.34)	**0.017**	0.05 (-0.08, 0.18)	0.444	1.46 (0.72, 2.20)	**<0.001**

Note: Models were adjusted with sex, age, occupation, monthly income, night-shift per month, intra-day rest time, exercise habits, weekly worktime and self-health evaluation.

Table S3 Associations of occupational stress with sleep quality in low and high hope classes.

**Hope**	Subjective sleep quality		Daytime dysfunction		Sleep latency		Sleep duration	
	β (95%CI)	*p* value	β (95%CI)	*p* value	β (95%CI)	*p* value	β (95%CI)	*p* value
**Below median (4.20)**								
Job requirement	0.18 (0.04, 0.31)	**0.011**	0.37 (0.25, 0.50)	**<0.001**	0.09 (-0.09, 0.28)	0.319	0.13 (-0.01, 0.27)	0.067
Organizational support	0.08 (-0.04, 0.21)	0.207	0.22 (0.09, 0.34)	**0.001**	0.03 (-0.14, 0.21)	0.689	0.12 (-0.01, 0.25)	0.072
Autonomy	-0.14 (-0.29, 0.01)	0.067	-0.11 (-0.26, 0.04)	0.137	-0.10 (-0.30, 0.11)	0.365	-0.16 (-0.32, 0.00)	0.052
Total score	0.23 (0.08, 0.38)	**0.004**	0.31 (0.16, 0.46)	**<0.001**	0.10 (-0.11, 0.31)	0.349	0.17 (0.01, 0.33)	**0.037**
**Equal or above median**								
Job requirement	0.21 (0.08, 0.35)	**0.002**	0.28 (0.16, 0.40)	**<0.001**	0.24 (0.07, 0.41)	**0.005**	0.28 (0.14, 0.41)	**<0.001**
Organizational support	0.14 (0.02, 0.25)	**0.018**	0.15 (0.05, 0.25)	**0.003**	0.21 (0.08, 0.35)	**0.002**	0.20 (0.09, 0.31)	**<0.001**
Autonomy	-0.21 (-0.36, -0.06)	**0.006**	-0.22 (-0.36, -0.09)	**0.001**	-0.25 (-0.43, -0.07)	**0.008**	-0.17 (-0.32, -0.02)	**0.029**
Total score	0.32 (0.14, 0.51)	**0.001**	0.40 (0.23, 0.57)	**<0.001**	0.34 (0.11, 0.57)	**0.003**	0.34 (0.16, 0.53)	**<0.001**
	Sleep efficiency		Sleep disturbances		Use of sleeping medications		PSQI	
	β (95%CI)	*p* value	β (95%CI)	*p* value	β (95%CI)	*p* value	β (95%CI)	*p* value
**Below median**								
Job requirement	0.08 (-0.08, 0.24)	0.352	0.28 (0.16, 0.40)	**<0.001**	-0.01 (-0.14, 0.11)	0.851	1.12 (0.55, 1.70)	**<0.001**
Organizational support	0.04 (-0.11, 0.19)	0.607	0.16 (0.05, 0.28)	**0.005**	0.05 (-0.06, 0.17)	0.385	0.71 (0.17, 1.24)	**0.010**
Autonomy	-0.08 (-0.26, 0.10)	0.366	-0.21 (-0.35, -0.07)	**0.003**	0.09 (-0.04, 0.23)	0.182	-0.70 (-1.35, -0.05)	**0.035**
Total score	0.08 (-0.10, 0.27)	0.363	0.33 (0.19, 0.47)	**<0.001**	-0.02 (-0.16, 0.12)	0.785	1.21 (0.55, 1.86)	**<0.001**
**Equal or above median**								
Job requirement	-0.05 (-0.18, 0.07)	0.404	0.16 (0.06, 0.27)	**0.003**	0.08 (-0.01, 0.17)	0.067	1.20 (0.68, 1.72)	**<0.001**
Organizational support	0.04 (-0.06, 0.15)	0.411	0.11 (0.02, 0.19)	**0.019**	0.02 (-0.06, 0.09)	0.659	0.87 (0.44, 1.29)	**<0.001**
Autonomy	-0.01 (-0.15, 0.13)	0.874	-0.14 (-0.26, -0.02)	**0.021**	0.02 (-0.08, 0.12)	0.678	-0.98 (-1.56, -0.41)	**0.001**
Total score	0.02 (-0.16, 0.19)	0.856	0.23 (0.08, 0.38)	**0.003**	0.04 (-0.08, 0.16)	0.545	1.69 (0.97, 2.41)	**<0.001**

Note: Models were adjusted with sex, age, occupation, monthly income, night-shift per month, intra-day rest time, exercise habits, weekly worktime and self-health evaluation.

Table S4 Associations of occupational stress with sleep quality in low and high resilience classes.

**Resilience**	Subjective sleep quality		Daytime dysfunction		Sleep latency		Sleep duration	
	β (95%CI)	*p* value	β (95%CI)	*p* value	β (95%CI)	*p* value	β (95%CI)	*p* value
**Below median (4.17)**								
Job requirement	0.23 (0.10, 0.36)	**0.001**	0.37 (0.25, 0.50)	**<0.001**	0.20 (0.02, 0.38)	**0.030**	0.16 (0.02, 0.30)	**0.026**
Organizational support	0.11 (-0.01, 0.23)	0.062	0.24 (0.13, 0.36)	**<0.001**	0.12 (-0.04, 0.28)	0.153	0.16 (0.04, 0.28)	**0.011**
Autonomy	-0.27 (-0.42, -0.12)	**0.001**	-0.13 (-0.28, 0.01)	0.072	-0.13 (-0.34, 0.07)	0.199	-0.23 (-0.39, -0.07)	**0.004**
Total score	0.37 (0.22, 0.53)	**<0.001**	0.35 (0.21, 0.50)	**<0.001**	0.23 (0.02, 0.44)	**0.034**	0.25 (0.08, 0.41)	0.003
**Equal or above median**								
Job requirement	0.14 (0.01, 0.28)	**0.041**	0.28 (0.15, 0.40)	**<0.001**	0.14 (-0.03, 0.31)	0.116	0.25 (0.11, 0.39)	**<0.001**

Organizational support	0.11 (-0.00, 0.23)	0.051	0.17 (0.06, 0.27)	**0.002**	0.15 (0.01, 0.29)	**0.035**	0.17 (0.06, 0.29)	**0.003**
Autonomy	-0.08 (-0.24, 0.07)	0.272	-0.20 (-0.34, -0.06)	**0.004**	-0.18 (-0.37, 0.00)	0.057	-0.09 (-0.25, 0.06)	0.229
Total score	0.12 (-0.07, 0.30)	0.210	0.35 (0.18, 0.52)	**<0.001**	0.14 (-0.09, 0.37)	0.231	0.24 (0.05, 0.42)	**0.012**
	Sleep efficiency		Sleep disturbances		Use of sleeping medications		PSQI	
	β (95%CI)	*p* value	β (95%CI)	*p* value	β (95%CI)	*p* value	β (95%CI)	*p* value
**Below median**								
Job requirement	0.10 (-0.06, 0.25)	0.221	0.32 (0.20, 0.44)	**<0.001**	0.02 (-0.10, 0.14)	0.732	1.40 (0.84, 1.96)	**<0.001**
Organizational support	0.09 (-0.04, 0.23)	0.172	0.19 (0.08, 0.30)	**0.001**	0.07 (-0.04, 0.17)	0.233	0.99 (0.49, 1.49)	**<0.001**
Autonomy	-0.07 (-0.24, 0.10)	0.425	-0.23 (-0.36, -0.09)	**0.001**	0.07 (-0.06, 0.21)	0.287	-0.99 (-1.62, -0.35)	**0.003**
Total score	0.08 (-0.10, 0.26)	0.361	0.39 (0.25, 0.53)	**<0.001**	0.02 (-0.13, 0.16)	0.796	1.69 (1.03, 2.35)	**<0.001**
**Equal or above median**								
Job requirement	-0.05 (-0.19, 0.08)	0.426	0.13 (0.02, 0.23)	**0.022**	0.03 (-0.06, 0.12)	0.502	0.91 (0.38, 1.44)	**0.001**
Organizational support	0.04 (-0.07, 0.15)	0.516	0.09 (0.00, 0.18)	**0.046**	-0.01 (-0.08, 0.06)	0.776	0.72 (0.28, 1.16)	**0.001**
Autonomy	-0.01 (-0.15, 0.14)	0.941	-0.12 (-0.24, -0.00)	**0.046**	0.06 (-0.04, 0.16)	0.225	-0.63 (-1.22, -0.04)	**0.037**
Total score	0.05 (-0.13, 0.23)	0.604	0.17 (0.03, 0.32)	**0.019**	-0.03 (-0.15, 0.08)	0.574	1.03 (0.32, 1.75)	**0.005**

Note: Models were adjusted with sex, age, occupation, monthly income, night-shift per month, intra-day rest time, exercise habits, weekly worktime and self-health evaluation.

Table S5 Associations of occupational stress with sleep quality in low and high potimism classes.

**Optimism**	Subjective sleep quality		Daytime dysfunction		Sleep latency		Sleep duration	
	β (95%CI)	*p* value	β (95%CI)	*p* value	β (95%CI)	*p* value	β (95%CI)	*p* value
**Below median (4.00)**								
Job requirement	-0.03 (-0.16, 0.09)	0.630	0.18 (0.09, 0.28)	**<0.001**	0.04 (-0.04, 0.13)	0.301	1.10 (0.63, 1.57)	**<0.001**
Organizational support	0.07 (-0.04, 0.17)	0.206	0.13 (0.05, 0.21)	**0.002**	-0.02 (-0.08, 0.05)	0.669	0.78 (0.39, 1.18)	**<0.001**
Autonomy	-0.02 (-0.16, 0.12)	0.765	-0.15 (-0.25, -0.04)	**0.009**	0.06 (-0.03, 0.15)	0.203	-0.71 (-1.24, -0.19)	**0.008**
Total score	0.06 (-0.11, 0.23)	0.469	0.24 (0.11, 0.37)	**<0.001**	-0.03 (-0.14, 0.09)	0.645	1.23 (0.58, 1.87)	**<0.001**
**Equal or above median**								
Job requirement	0.17 (0.05, 0.30)	**0.006**	0.27 (0.16, 0.38)	**<0.001**	0.18 (0.02, 0.34)	**0.025**	0.28 (0.15, 0.40)	**<0.001**
Organizational support	0.13 (0.03, 0.23)	**0.013**	0.17 (0.08, 0.26)	**<0.001**	0.15 (0.02, 0.28)	**0.027**	0.16 (0.05, 0.26)	**0.004**
Autonomy	-0.11 (-0.24, 0.03)	0.122	-0.18 (-0.30, -0.06)	**0.003**	-0.16 (-0.33, 0.01)	**0.064**	-0.15 (-0.29, -0.01)	**0.033**
Total score	0.18 (0.01, 0.35)	**0.036**	0.30 (0.16, 0.45)	**<0.001**	0.15 (-0.06, 0.37)	0.156	0.31 (0.14, 0.48)	**<0.001**
	Sleep efficiency		Sleep disturbances		Use of sleeping medications		PSQI	
	β (95%CI)	*p* value	β (95%CI)	*p* value	β (95%CI)	*p* value	β (95%CI)	*p* value
**Below median**								
Job requirement	0.10 (-0.07, 0.27)	0.259	0.31 (0.17, 0.44)	**<0.001**	0.03 (-0.12, 0.17)	0.728	1.36 (0.70, 2.03)	**<0.001**
Organizational support	0.04 (-0.11, 0.20)	0.598	0.15 (0.02, 0.27)	**0.023**	0.12 (-0.01, 0.25)	0.074	0.94 (0.33, 1.54)	**0.002**
Autonomy	-0.07 (-0.27, 0.12)	0.447	-0.22 (-0.38, -0.07)	**0.005**	0.04 (-0.12, 0.20)	0.593	-1.09 (-1.83, -0.34)	**0.004**
Total score	0.07 (-0.12, 0.27)	0.462	0.36 (0.21, 0.52)	**<0.001**	0.05 (-0.11, 0.21)	0.525	1.72 (0.97, 2.47)	**<0.001**
**Equal or above median**								
Job requirement	0.24 (0.09, 0.39)	**0.002**	0.42 (0.27, 0.56)	**<0.001**	0.16 (-0.04, 0.36)	0.126	0.12 (-0.04, 0.27)	0.147
Organizational support	0.09 (-0.04, 0.23)	0.184	0.24 (0.11, 0.38)	**<0.001**	0.11 (-0.07, 0.29)	0.241	0.18 (0.04, 0.32)	**0.012**
Autonomy	-0.27 (-0.44, -0.11)	**0.001**	-0.20 (-0.37, -0.03)	**0.023**	-0.20 (-0.43, 0.02)	0.081	-0.16 (-0.34, 0.01)	0.068
Total score	0.37 (0.20, 0.54)	**<0.001**	0.43 (0.26, 0.60)	**<0.001**	0.26 (0.03, 0.49)	**0.026**	0.17 (-0.01, 0.35)	0.066

Note: Models were adjusted with sex, age, occupation, monthly income, night-shift per month, intra-day rest time, exercise habits, weekly worktime and self-health evaluation.

Table S6 Results of mediation analysis of self-efficacy.

**Self-efficacy**		Subjective sleep quality		Daytime dysfunction		Sleep latency		Sleep duration	
		β (95%CI)	*p* value	β (95%CI)	*p* value	β (95%CI)	*p* value	β (95%CI)	*p* value
Job requirement	ACME	0.028 (0.011, 0.049)	**<0.001**	0.032 (0.014, 0.054)	**<0.001**	0.030 (0.011, 0.054)	**<0.001**	0.014 (0.003, 0.030)	**0.008**
	Proportion	0.134 (0.053, 0.281)	**<0.001**	0.089 (0.039, 0.155)	**<0.001**	0.163 (0.056, 0.512)	**0.004**	0.063 (0.012, 0.157)	**0.008**
Organizational support	ACME	0.059 (0.033, 0.088)	**<0.001**	0.065 (0.039, 0.093)	**<0.001**	0.061 (0.026, 0.097)	**<0.001**	0.023 (-0.002, 0.050)	0.080
	Proportion	0.367 (0.178, 0.778)	**<0.001**	0.257 (0.149, 0.413)	**<0.001**	0.330 (0.129, 0.792)	**<0.001**	0.123 (-0.012, 0.327)	0.080
Autonomy	ACME	-0.047 (-0.075, -0.025)	**<0.001**	-0.058 (-0.087, -0.033)	**<0.001**	-0.050 (-0.082, -0.024)	**<0.001**	-0.024 (-0.046, -0.005)	**0.015**
	Proportion	0.240 (0.114, 0.551)	**<0.001**	0.283 (0.151, 0.596)	**0.001**	0.266 (0.104, 0.912)	**0.005**	0.134 (0.025, 0.405)	**0.018**
Total score	ACME	0.053 (0.028, 0.084)	**<0.001**	0.061 (0.035, 0.092)	**<0.001**	0.059 (0.028, 0.095)	**<0.001**	0.026 (0.004, 0.052)	**0.021**
	Proportion	0.185 (0.093, 0.344)	**<0.001**	0.155 (0.087, 0.252)	**<0.001**	0.274 (0.106, 0.911)	**0.008**	0.101 (0.015, 0.261)	**0.021**

		Sleep efficiency		Sleep disturbances		Use of sleeping medications		PSQI	
		β (95%CI)	*p* value	β (95%CI)	*p* value	β (95%CI)	*p* value	β (95%CI)	*p* value
Job requirement	ACME	0.012 (0.001, 0.028)	**0.035**	0.019 (0.007, 0.035)	**<0.001**	0.011 (0.002, 0.023)	**0.010**	0.148 (0.061, 0.245)	**<0.001**
	Proportion	0.145 (-3.000, 2.989)	0.584	0.078 (0.029, 0.158)	**<0.001**	0.201 (-2.132, 2.814)	0.306	0.114 (0.050, 0.205)	**<0.001**
Organizational support	ACME	0.025 (-0.002, 0.054)	0.068	0.038 (0.016, 0.061)	**<0.001**	0.025 (0.005, 0.046)	**0.014**	0.296 (0.188, 0.417)	**<0.001**
	Proportion	0.349 (-2.456, 3.459)	0.190	0.228 (0.094, 0.443)	**<0.001**	0.487 (-4.877, 6.451)	0.220	0.283 (0.169, 0.447)	**<0.001**
Autonomy	ACME	-0.021 (-0.044, -0.002)	**0.034**	-0.033 (-0.054, -0.015)	**<0.001**	-0.022 (-0.039, -0.007)	**0.004**	-0.254 (-0.381, -0.150)	**<0.001**
	Proportion	0.250 (-4.039, 4.836)	0.413	0.171 (0.075, 0.369)	**<0.001**	-0.330 (-4.564, 4.201)	0.279	0.265 (0.145, 0.515)	**<0.001**
Total score	ACME	0.023 (0.000, 0.049)	**0.044**	0.035 (0.016, 0.059)	**<0.001**	0.023 (0.005, 0.043)	**0.008**	0.281 (0.163, 0.423)	**<0.001**
	Proportion	0.234 (-2.549, 2.997)	0.300	0.110 (0.049, 0.208)	**<0.001**	0.303 (-6.934, 6.951)	0.742	0.179 (0.103, 0.301)	**<0.001**

Note: Models were adjusted with sex, age, occupation, monthly income, night-shift per month, intra-day rest time, exercise habits, weekly worktime and self-health evaluation.

Table S7 Results of mediation analysis of hope.

**Hope**		Subjective sleep quality		Daytime dysfunction		Sleep latency		Sleep duration	
		β (95%CI)	*p* value	β (95%CI)	*p* value	β (95%CI)	*p* value	β (95%CI)	*p* value
Job requirement	ACME	0.049 (0.027, 0.074)	**<0.001**	0.056 (0.033, 0.084)	**<0.001**	0.047 (0.023, 0.076)	**<0.001**	0.025 (0.008, 0.045)	**0.002**
	Proportion	0.232 (0.122, 0.452)	**<0.001**	0.155 (0.093, 0.242)	**<0.001**	0.257 (0.113, 0.829)	**0.003**	0.114 (0.036, 0.258)	**0.002**
Organizational support	ACME	0.080 (0.051, 0.112)	**<0.001**	0.090 (0.061, 0.122)	**<0.001**	0.072 (0.036, 0.112)	**<0.001**	0.035 (0.007, 0.065)	**0.011**
	Proportion	0.494 (0.274, 1.013)	**<0.001**	0.356 (0.228, 0.552)	**<0.001**	0.390 (0.174, 0.960)	**<0.001**	0.190 (0.035, 0.426)	**0.011**
Autonomy	ACME	-0.062 (-0.094, -0.036)	**<0.001**	-0.077 (-0.111, -0.047)	**<0.001**	-0.059 (-0.094, -0.030)	**<0.001**	-0.033 (-0.058, -0.012)	**<0.001**
	Proportion	0.320 (0.160, 0.698)	**0.001**	0.377 (0.216, 0.744)	**<0.001**	0.314 (0.134, 1.071)	**0.007**	0.186 (0.065, 0.508)	**<0.001**
Total score	ACME	0.074 (0.043, 0.108)	**<0.001**	0.087 (0.055, 0.124)	**<0.001**	0.073 (0.037, 0.113)	**<0.001**	0.039 (0.013, 0.069)	**0.001**
	Proportion	0.257 (0.142, 0.472)	**<0.001**	0.221 (0.138, 0.341)	**<0.001**	0.334 (0.141, 1.135)	**0.007**	0.152 (0.050, 0.340)	**0.001**

		Sleep efficiency		Sleep disturbances		Use of sleeping medications		PSQI	
		β (95%CI)	*p* value	β (95%CI)	*p* value	β (95%CI)	*p* value	β (95%CI)	*p* value
Job requirement	ACME	0.011 (-0.006, 0.029)	0.196	0.030 (0.014, 0.050)	**<0.001**	0.010 (-0.002, 0.025)	0.098	0.227 (0.130, 0.341)	**<0.001**
	Proportion	0.121 (-3.107, 3.935)	0.640	0.123 (0.057, 0.228)	**<0.001**	0.178 (-2.540, 3.016)	0.393	0.177 (0.101, 0.289)	**<0.001**
Organizational support	ACME	0.014 (-0.016, 0.043)	0.364	0.046 (0.022, 0.071)	**<0.001**	0.016 (-0.006, 0.038)	0.148	0.354 (0.233, 0.486)	**<0.001**
	Proportion	0.176 (-1.485, 2.762)	0.454	0.280 (0.128, 0.531)	**<0.001**	0.309 (-2.968, 4.063)	0.324	0.336 (0.205, 0.531)	**<0.001**
Autonomy	ACME	-0.013 (-0.036, 0.008)	0.236	-0.039 (-0.063, -0.020)	**<0.001**	-0.016 (-0.034, -0.000)	**0.047**	-0.299 (-0.431, -0.181)	**<0.001**
	Proportion	0.146 (-2.501, 3.141)	0.544	0.204 (0.094, 0.420)	**<0.001**	-0.246 (-4.190, 3.126)	0.306	0.314 (0.178, 0.595)	**<0.001**
Total score	ACME	0.015 (-0.011, 0.042)	0.248	0.044 (0.022, 0.071)	**<0.001**	0.017 (-0.002, 0.038)	0.084	0.350 (0.220, 0.503)	**<0.001**
	Proportion	0.146 (-1.825, 2.182)	0.437	0.140 (0.067, 0.254)	**<0.001**	0.195 (-6.119, 5.905)	0.739	0.225 (0.134, 0.355)	**<0.001**

Note: Models were adjusted with sex, age, occupation, monthly income, night-shift per month, intra-day rest time, exercise habits, weekly worktime and self-health evaluation.

Table S8 Results of mediation analysis of resilience.

**Resilience**		Subjective sleep quality		Daytime dysfunction		Sleep latency		Sleep duration	
		β (95%CI)	*p* value	β (95%CI)	*p* value	β (95%CI)	*p* value	β (95%CI)	*p* value
Job requirement	ACME	0.036 (0.017, 0.058)	**<0.001**	0.047 (0.025, 0.072)	**<0.001**	0.031 (0.012, 0.056)	**0.002**	0.016 (0.002, 0.033)	**0.021**
	Proportion	0.171 (0.079, 0.351)	**<0.001**	0.128 (0.069, 0.206)	**<0.001**	0.166 (0.056, 0.538)	**0.004**	0.071 (0.009, 0.175)	**0.021**
Organizational support	ACME	0.065 (0.037, 0.095)	**<0.001**	0.084 (0.056, 0.114)	**<0.001**	0.050 (0.015, 0.087)	**0.004**	0.021 (-0.007, 0.049)	0.129
	Proportion	0.399 (0.207, 0.817)	**<0.001**	0.330 (0.211, 0.509)	**<0.001**	0.271 (0.077, 0.683)	**0.005**	0.115 (-0.042, 0.316)	0.129
Autonomy	ACME	-0.031 (-0.055, -0.012)	**0.002**	-0.043 (-0.071, -0.017)	**0.001**	-0.027 (-0.050, -0.008)	**<0.001**	-0.015 (-0.032, -0.003)	**0.010**
	Proportion	0.156 (0.058, 0.369)	**0.002**	0.210 (0.084, 0.446)	**0.001**	0.137 (0.039, 0.522)	**0.006**	0.082 (0.016, 0.254)	**0.012**
Total score	ACME	0.046 (0.023, 0.075)	**<0.001**	0.063 (0.034, 0.096)	**<0.001**	0.041 (0.015, 0.072)	**<0.001**	0.021 (0.003, 0.044)	**0.017**
	Proportion	0.162 (0.078, 0.315)	**<0.001**	0.159 (0.086, 0.256)	**<0.001**	0.188 (0.062, 0.676)	**0.005**	0.082 (0.012, 0.210)	**0.017**

		Sleep efficiency		Sleep disturbances		Use of sleeping medications		PSQI	
		β (95%CI)	*p* value	β (95%CI)	*p* value	β (95%CI)	*p* value	β (95%CI)	*p* value
Job requirement	ACME	0.009 (-0.005, 0.025)	0.220	0.026 (0.012, 0.042)	**<0.001**	0.014 (0.004, 0.028)	**0.007**	0.177 (0.091, 0.275)	**<0.001**
	Proportion	0.096 (-2.583, 2.449)	0.660	0.106 (0.046, 0.199)	**<0.001**	0.248 (-3.570, 3.970)	0.321	0.137 (0.070, 0.233)	**<0.001**
Organizational support	ACME	0.012 (-0.017, 0.041)	0.400	0.045 (0.022, 0.070)	**<0.001**	0.027 (0.007, 0.049)	**0.01**	0.304 (0.191, 0.429)	**<0.001**
	Proportion	0.164 (-1.531, 2.217)	0.480	0.270 (0.123, 0.517)	**<0.001**	0.548 (-5.809, 6.567)	0.226	0.287 (0.174, 0.461)	**<0.001**
Autonomy	ACME	-0.007 (-0.021, 0.005)	0.236	-0.023 (-0.041, -0.008)	**0.001**	-0.013 (-0.027, -0.003)	**0.001**	-0.159 (-0.268, -0.063)	**0.001**
	Proportion	0.081 (-1.507, 1.758)	0.539	0.119 (0.042, 0.257)	**0.001**	-0.198 (-2.913, 2.451)	0.271	0.166 (0.068, 0.340)	**0.001**

Total score	ACME	0.011 (-0.008, 0.032)	0.262	0.034 (0.015, 0.056)	**<0.001**	0.020 (0.005, 0.038)	**0.008**	0.236 (0.126, 0.370)	**<0.001**
	Proportion	0.102 (-1.370, 1.781)	0.450	0.106 (0.049, 0.198)	**<0.001**	0.251 (-6.248, 5.251)	0.734	0.151 (0.079, 0.261)	**<0.001**

Note: Models were adjusted with sex, age, occupation, monthly income, night-shift per month, intra-day rest time, exercise habits, weekly worktime and self-health evaluation.

Table S9 Results of mediation analysis of optimism.

**Optimism**		Subjective sleep quality		Daytime dysfunction		Sleep latency		Sleep duration	
		β (95%CI)	*p* value	β (95%CI)	*p* value	β (95%CI)	*p* value	β (95%CI)	*p* value
Job requirement	ACME	0.054 (0.031, 0.081)	**<0.001**	0.056 (0.033, 0.082)	**<0.001**	0.057 (0.029, 0.090)	**<0.001**	0.032 (0.013, 0.056)	**<0.001**
	Proportion	0.260 (0.139, 0.491)	**<0.001**	0.154 (0.089, 0.240)	**<0.001**	0.313 (0.137, 0.932)	**0.003**	0.146 (0.055, 0.320)	**<0.001**
Organizational support	ACME	0.098 (0.061, 0.136)	**<0.001**	0.094 (0.058, 0.132)	**<0.001**	0.098 (0.048, 0.149)	**<0.001**	0.046 (0.009, 0.084)	**0.015**
	Proportion	0.606 (0.332, 1.248)	**<0.001**	0.372 (0.221, 0.591)	**<0.001**	0.529 (0.235, 1.281)	**<0.001**	0.253 (0.050, 0.573)	**0.015**
Autonomy	ACME	-0.045 (-0.071, -0.023)	**<0.001**	-0.050 (-0.079, -0.026)	**<0.001**	-0.047 (-0.077, -0.022)	**<0.001**	-0.028 (-0.050, -0.011)	**0.002**
	Proportion	0.227 (0.108, 0.517)	**<0.001**	0.244 (0.118, 0.504)	**0.002**	0.246 (0.097, 0.841)	**0.009**	0.159 (0.057, 0.461)	**0.003**
Total score	ACME	0.065 (0.037, 0.097)	**<0.001**	0.069 (0.041, 0.103)	**<0.001**	0.070 (0.036, 0.111)	**<0.001**	0.040 (0.016, 0.068)	**<0.001**
	Proportion	0.228 (0.120, 0.422)	**<0.001**	0.174 (0.102, 0.283)	**<0.001**	0.322 (0.135, 1.050)	**0.007**	0.155 (0.060, 0.353)	**<0.001**
		Sleep efficiency		Sleep disturbances		Use of sleeping medications		PSQI	
		β (95%CI)	*p* value	β (95%CI)	*p* value	β (95%CI)	*p* value	β (95%CI)	*p* value
Job requirement	ACME	0.020 (0.000, 0.043)	0.050	0.029 (0.013, 0.048)	**<0.001**	0.015 (0.000, 0.031)	**0.047**	0.264 (0.165, 0.384)	**<0.001**
	Proportion	0.250 (-5.387, 5.346)	0.596	0.119 (0.050, 0.225)	**<0.001**	0.251 (-3.217, 3.891)	0.337	0.205 (0.122, 0.329)	**<0.001**
Organizational support	ACME	0.031 (-0.007, 0.070)	0.109	0.045 (0.013, 0.078)	**0.006**	0.026 (-0.002, 0.056)	0.072	0.439 (0.283, 0.603)	**<0.001**
	Proportion	0.424 (-3.053, 5.323)	0.232	0.272 (0.075, 0.570)	**0.006**	0.505 (-6.551, 6.943)	0.284	0.418 (0.254, 0.666)	**<0.001**
Autonomy	ACME	-0.015 (-0.034, 0.000)	0.052	-0.026 (-0.044, -0.011)	**<0.001**	-0.014 (-0.028, -0.002)	**0.022**	-0.226 (-0.352, -0.118)	**<0.001**
	Proportion	0.179 (-2.541, 3.050)	0.428	0.134 (0.056, 0.292)	**<0.001**	-0.208 (-2.985, 2.264)	0.269	0.235 (0.118, 0.458)	**<0.001**
Total score	ACME	0.022 (-0.001, 0.048)	0.060	0.034 (0.015, 0.058)	**<0.001**	0.019 (0.001, 0.039)	**0.042**	0.317 (0.191, 0.461)	**<0.001**
	Proportion	0.221 (-2.560, 2.845)	0.298	0.110 (0.045, 0.205)	**<0.001**	0.219 (-5.448, 5.893)	0.746	0.205 (0.120, 0.328)	**<0.001**

Note: Models were adjusted with sex, age, occupation, monthly income, night-shift per month, intra-day rest time, exercise habits, weekly worktime and self-health evaluation.

Table S10 Results of mediation analysis of total score of PsyCap.

**Total score of PsyCap**		Subjective sleep quality		Daytime dysfunction		Sleep latency		Sleep duration	
		β (95%CI)	*p* value	β (95%CI)	*p* value	β (95%CI)	*p* value	β (95%CI)	*p* value
Job requirement	ACME	0.055 (0.032, 0.083)	**<0.001**	0.063 (0.038, 0.092)	**<0.001**	0.054 (0.029, 0.086)	**<0.001**	0.028 (0.010, 0.050)	**0.002**
	Proportion	0.265 (0.145, 0.519)	**<0.001**	0.176 (0.106, 0.266)	**<0.001**	0.294 (0.133, 0.897)	**0.006**	0.127 (0.045, 0.281)	**0.002**
Organizational support	ACME	0.105 (0.069, 0.143)	**<0.001**	0.117 (0.082, 0.155)	**<0.001**	0.098 (0.053, 0.145)	**<0.001**	0.042 (0.009, 0.078)	**0.014**
	Proportion	0.645 (0.369, 1.297)	**<0.001**	0.464 (0.303, 0.711)	**<0.001**	0.532 (0.245, 1.229)	**0.001**	0.230 (0.044, 0.528)	**0.014**
Autonomy	ACME	-0.064 (-0.094, -0.038)	**<0.001**	-0.078 (-0.111, -0.048)	**<0.001**	-0.062 (-0.098, -0.033)	**<0.001**	-0.034 (-0.059, -0.014)	**<0.001**
	Proportion	0.324 (0.172, 0.711)	**<0.001**	0.383 (0.219, 0.751)	**<0.001**	0.322 (0.136, 1.082)	**0.009**	0.196 (0.072, 0.531)	**0.001**
Total score	ACME	0.080 (0.049, 0.117)	**<0.001**	0.096 (0.063, 0.135)	**<0.001**	0.082 (0.044, 0.124)	**<0.001**	0.042 (0.015, 0.072)	**0.004**
	Proportion	0.281 (0.161, 0.527)	**<0.001**	0.243 (0.156, 0.374)	**<0.001**	0.378 (0.170, 1.326)	**0.005**	0.164 (0.058, 0.367)	**0.004**
		Sleep efficiency		Sleep disturbances		Use of sleeping medications		PSQI	
		β (95%CI)	*p* value	β (95%CI)	*p* value	β (95%CI)	*p* value	β (95%CI)	*p* value
Job requirement	ACME	0.018 (0.000, 0.038)	**0.046**	0.035 (0.019, 0.055)	**<0.001**	0.018 (0.005, 0.034)	**0.007**	0.271 (0.164, 0.397)	**<0.001**
	Proportion	0.221 (-4.990, 4.799)	0.588	0.146 (0.075, 0.262)	**<0.001**	0.322 (-4.336, 4.323)	0.311	0.212 (0.128, 0.340)	**<0.001**
Organizational support	ACME	0.030 (-0.005, 0.066)	0.096	0.062 (0.032, 0.094)	**<0.001**	0.034 (0.008, 0.063)	**0.011**	0.488 (0.336, 0.650)	**<0.001**
	Proportion	0.405 (-2.148, 4.295)	0.213	0.376 (0.186, 0.713)	**<0.001**	0.695 (-7.663, 8.429)	0.225	0.464 (0.298, 0.724)	**<0.001**
Autonomy	ACME	-0.020 (-0.043, 0.000)	0.051	-0.042 (-0.065, -0.023)	**<0.001**	-0.023 (-0.042, -0.008)	**0.001**	-0.323 (-0.466, -0.200)	**<0.001**
	Proportion	0.226 (-4.154, 3.760)	0.440	0.221 (0.110, 0.445)	**<0.001**	-0.359 (-5.349, 4.575)	0.276	0.334 (0.194, 0.625)	**<0.001**
Total score	ACME	0.025 (-0.001, 0.054)	0.062	0.050 (0.027, 0.078)	**<0.001**	0.028 (0.008, 0.052)	**0.003**	0.404 (0.262, 0.573)	**<0.001**
	Proportion	0.253 (-2.560, 3.968)	0.289	0.160 (0.085, 0.276)	**<0.001**	0.363 (-9.392, 9.320)	0.737	0.261 (0.166, 0.414)	**<0.001**

Note: Models were adjusted with sex, age, occupation, monthly income, night-shift per month, intra-day rest time, exercise habits, weekly worktime and self-health evaluation.
